# Platinum nanocluster catalysts supported on Marimo carbon *via* scalable dry deposition synthesis†

**DOI:** 10.1039/d1ra07717a

**Published:** 2021-12-08

**Authors:** Naoyuki Hirata, Yui Katsura, Hiroyuki Gunji, Masahide Tona, Keizo Tsukamoto, Mika Eguchi, Toshihiro Ando, Atsushi Nakajima

**Affiliations:** Ayabo Co., Ltd. 1 Hosogute Fukukama-cho Anjo Aichi 446-0052 Japan keizo@ayabo.com; College of Engineering, Ibaraki University 4-12-1 Nakanarusawa Hitachi Ibaraki 316-8511 Japan mika.eguchi.m@vc.ibaraki.ac.jp; National Institute for Materials Science Tsukuba Ibaraki 305-0044 Japan ANDO.Toshihiro@nims.go.jp; Department of Chemistry, Keio University 3-14-1 Hiyoshi Kohoku-ku Yokohama Kanagawa 223-8522 Japan nakajima@chem.keio.ac.jp

## Abstract

The development of efficient fuel cells greatly promotes reducing the consumption of fossil energy, and it is crucial to enhance the platinum (Pt) catalytic activity by optimizing both the nanoparticle size and support effect. In this study, we generate a smaller and uniform size of naked Pt nanocluster (NC) catalysts *via* a dry process in the gas phase, and using the direct powder embedded trapping method, the Pt NCs are *spatioselectively* supported on Marimo carbon (MC) that comprises a high density of carbon nanofilaments. At a minimum Pt loading of 0.05 mg_Pt_ cm^−2^ for both the anode and cathode in a single cell configuration, a membrane-electrode assembly (MEA) formed by the dry-deposition Pt-NC(*d*)/MC exhibits superior power density (rated) of 0.105 g_Pt_ kW^−1^ at a current density of 1.2 A cm^−2^, which is better output power density than the conventional MEA formed by Pt catalysts *via* a wet process. The origin of the improved performance is investigated using transmission electron microscopy; dry-deposition Pt catalysts that are monodispersely loaded on the outer surface of MC can facilitate not only the gas reaction but also leaving the generated water. The present results demonstrate that the dry deposition of Pt NCs on MC can be used as a scalable catalyst synthesis method to reduce the Pt loading.

## Abstract 2

The development of efficient fuel cells greatly promotes reducing the consumption of fossil energy, and it is crucial to enhance the platinum (Pt) catalytic activity by optimizing both the nanoparticle size and support effect.

## Graphical abstract

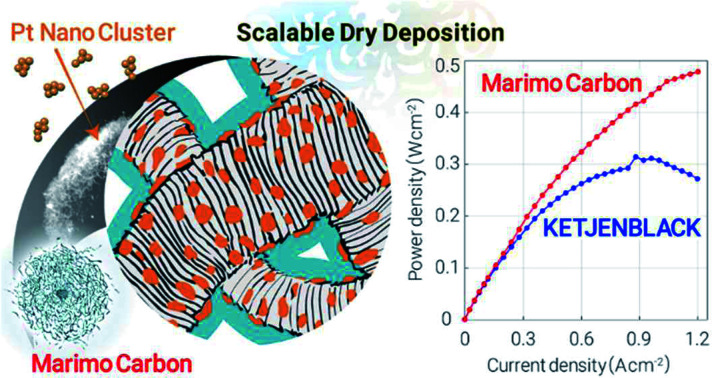

## Introduction

To overcome the global challenges of the shortage of fossil fuels and environmental pollution, it is crucial to promote a clean energy technology.^1^ Hydrogen is a promising candidate as the energy source, allowing us to build the hydrogen cycle with fuel cells (FCs). Among various FCs, polymer electrolyte FCs (PEFCs) are one of the most promising renewable energy technologies for a vehicle's electric motor, where the chemical energy of hydrogen can be cleanly converted to electricity without carbon dioxide emissions.^2^ Among all the monometallic catalysts for the oxygen reduction reaction (ORR) at the cathode of the PEFC, platinum (Pt) nanoparticles (NPs) have been the most widely studied for decades. However, it is highly required to substantially improve the ORR activity to reduce expensive Pt usage^3–16^ because the slow ORR kinetics result in an overpotential due to the relatively strong binding of oxygen species.^17^

There are two approaches for improving the ORR activity; alloying and support effect. Alloying Pt NPs with transition metal or lanthanide elements^6–12,18–21^ has been extensively explored to optimize the electronic properties of catalysts. The second approach is to design the interaction between catalysts and supports including well-architected nanostructures. Electrocatalytic performance can be maximized by optimizing surface immobilization sites and designing optimal nanostructures for supplying reaction gas and discharging products.^22–31^

**Fig. 1:**
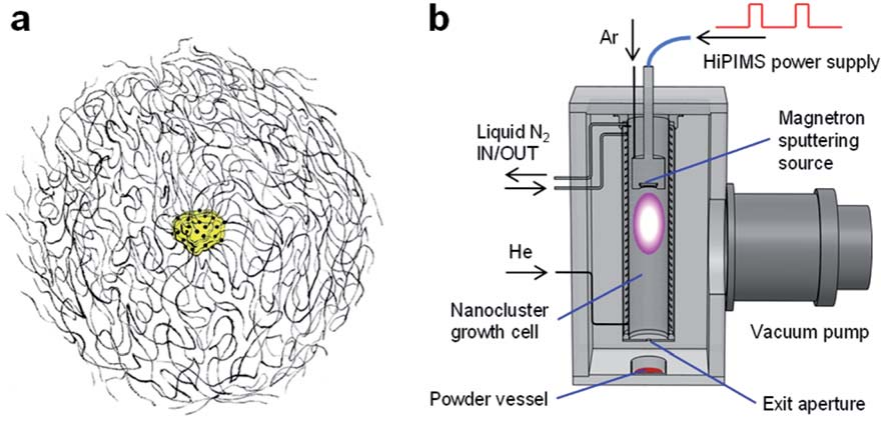
(a) Schematic structural view of Marimo carbon (MC). (b) Schematic setup for depositing Pt nanoclusters (NCs) on material powder. Pt NCs, generated by high-power impulse magnetron sputtering (HiPIMS), are deposited on the target powder, while the powder is stirred downstream of the NC beam with a magnetic stirrer located outside the vacuum chamber. This method is called the direct powder embedded trapping (DiPET) method.

In fabricating well-architected nanostructures, Marimo carbon (MC) has high potential as a carbon material to support Pt catalysts for FCs,^32,33^ MC is illustrated in Fig. 1(a), consists of high-density carbon nanofilaments (CNFs) and has a spherical secondary shape, where an oxidized metal particle is centered at the core.^34^ Since the CNFs are formed by stacking multiple rolled graphene sheets like a cup shape, the CNF surface is composed of edges of the graphene sheets, which can efficiently support metal catalysts as a support. In particular, when Pt catalysts are spatioselectively supported on the outer surface, the spherical shape with high density CNFs can facilitate high gas diffusivity in the preformed layers with ionomers.

In the Pt catalysts loading on carbon support materials, a wet process is usually used, in which hexachloroplatinic acid (H_2_PtCl_6_) is reduced in a solution with dispersed carbon powder. Since the Pt catalysts are generated by atom aggregations, the wet process allows the Pt NPs to be formed in internally deep pores as well as the support surface, and the buried Pt NPs may not be contributed to the catalytic activity owing to limited supplies of reactant gases.^35,36^ In contrast, *preformed* Pt nanoclusters (NC(*d*)s) *via* a dry process in the gas phase can be preferably immobilized at the surface because of the formation of an abrupt interface between Pt NC(*d*)s and carbon materials with minimizing the buried Pt.^37^ Moreover, scalable NC generation can be recently achievable by the development of an intensive NC source with high-power impulse magnetron sputtering (HiPIMS).^38,39^

The catalytic activity for the ORR has often been examined by liquid half-cells, which are well suited for laboratory-scale studies due to the small amount of catalysts. Although these studies have revealed detailed catalytic information and many fundamental bottlenecks at the electrochemical interface, it is necessary to extend the FC performance evaluation to investigate well-defined catalysts at a high current under real device conditions of a single cell.^22,40^ Furthermore, while a demanding task of FCs with lower Pt loading is more challenging (<0.1 mg_Pt_ cm^−2^ per a membrane-electrode assembly (MEA)), overall FC performance will become susceptible to accessibility of protons and gases to Pt catalysts due to locally restricted transport, which results in the loss at high power region.^41^

In this study, to develop an efficient PEFC with increasing the Pt utilization efficiency, smaller and more uniformly sized Pt NC catalysts are generated by a dry process in the gas phase,^38,39,42^ and the Pt NC(*d*) catalysts are directly embedded on MC powder, forming dry-deposition Pt-NC(*d*)/MC. By measuring the output power density of the FC with a single cell by fabricating a MEA of size 25 cm^2^, the power density of the MEA formed by Pt-NC(*d*)/MC, at minimum Pt loading of 0.05 mg_Pt_ cm^−2^ for both anode and cathode (0.1 mg_Pt_ cm^−2^ in total), is observed to be better than that formed by Pt NP catalysts loaded on MC through a wet process (Pt-NP(*w*)/MC). In addition to the FC performance by Pt catalysts on carbon black nanomaterials, the origin of the improved performance of Pt-NC(*d*)/MC (superior power density (rated) of 0.105 g_Pt_ kW^−1^ at a current density of 1.2 A cm^−2^) is discussed with transmission electron microscopy (TEM) images; dry-deposition Pt catalysts that are monodispersely loaded on the outer surface of MC can facilitate not only the gas reaction but also leaving the generated water, exhibiting superior power generation compared to Pt-NP(*w*)/MC.

## Experimental

Four types of Pt catalysts were prepared: two carbon support materials of MC and carbon black nanomaterials (Ketjenblack; KB) with two different loading methods, namely, gas-phase embedded trapping for Pt NCs and conventional liquid synthesis for Pt NPs. MC was synthesized by reported procedures,^32^ while commercially available carbon nanomaterials of KB (Lion Specialty Chemicals Co., Ltd., EC300J (ECP)) were used without further purification.

### MC synthesis

MC was synthesized by thermal chemical vapor deposition of methane (CH_4_) at 5 wt% Ni/O–diamond as a catalyst, where the Ni/O–diamond was initially prepared in oxidation pretreatment of supported Ni metal nanoparticles by incipient wetness impregnation to diamond particles.^32^ CH_4_ was flowed at a rate of 740 mL min^−1^ at 823 K under atmospheric pressure, and was decomposed in a flow reactor. Using 500 mg of the Ni/O–diamond catalyst in each run, 2 g scale of the MC in total was synthesized in several runs, where each run was 5 h. Note that Ni dissolution from MC is negligible due to complete coverage by carbon nanofilaments; indeed, Ni was below the detection limit with inductively coupled plasma optical emission spectrometry.

### Gas-phase synthesis of Pt NCs

The experimental procedure of Pt NC deposition on MC was very similar to that of the direct liquid embedded trapping (DiLET) method,^43,44^ except that powder material was used for trapping instead of liquid, as illustrated in Fig. 1(b). Briefly, Pt NCs were generated by the intensive NC source^38,39^ based on HiPIMS powered by a modulated-pulse power supply (Zpulser LLC, AXIA-3X) combined with a gas flow reactor. A disk Pt target (50.8 mm*ϕ* in diameter, 99.99% purity) was sputtered with a magnetron sputtering cathode (Angstrom Sciences Inc., ONYX-MAGII) inside a NC growth cell cooled by liquid nitrogen. In the vicinity of the Pt target, argon gas (Ar; 99.9995% purity) was introduced to enhance the sputtering rate, and atomic Pt neutrals and ions were produced by HiPIMS, where the peak power of discharge and sputtering repetition rate were typically 0.2–0.3 kW and 110 Hz, respectively. The generated atomic Pt species grew into Pt NCs by the buffer-gas cooling of helium gas (He; 99.9998% purity). The flow rates of Ar and He gases were controlled by individual mass flow controllers to keep pressure in the cell to be 5–20 Pa; typical flow rates for Ar and He were 110 and 200 sccm, respectively. The produced Pt NC ions and neutrals were expanded into a vacuum chamber through an aperture (12 mm*ϕ* in diameter), which was evacuated by a turbomolecular pump for high throughput (2000 L s^−1^) to maintain a pressure of <0.1 Pa during the operation.

### Pt NC deposition on MC

MC powder (2.5 g) in a glass laboratory dish (90 mm*ϕ* in diameter) was placed 100 mm downstream of the aperture of the NC growth cell. The generated Pt NCs were dispersed over the MC powder under gentle stirring with a magnetic stirrer (∼80 rpm), which is called the direct powder embedded trapping (DiPET) method (Fig. 1(b)). After trapping the Pt NCs for 50 h, the Pt NC on MC powder generated by the dry process (Pt-NC(*d*)/MC) was removed from the chamber by transferring it using a sealed glass vessel.

During the NC deposition, the stirring operation reduces the amount of the MC powder to about 80% due to the scattering out of the glass laboratory dish (20%) in the chamber, and approximately 8 wt% of Pt NC is deposited therein as products. 10–5% Pt sputtered from the target is deposited on the MC powder, and the remaining 80% is recovered from the inner wall of the growth cell. Pt catalyst is robust against oxidation under ambient conditions, judging from X-ray photoelectron spectroscopy and X-ray absorption fine structure.^42^ It should be noted that the sputtering conditions for Pt NC generation were optimized with another HiPIMS NC source, which was equipped with a quadrupole mass spectrometer (Extrel CMS LLC, MAX-16000),^38,39^ and the same cell pressures under similar sputtering conditions were used.

### Pt NP loading on MC or KB

Pt NPs were loaded on MC or KB in liquid phase synthesis under a wet process. As previously reported,^32,33,45^ an aqueous solution of chloroplatinic acid and citric acid was added and mixed in an aqueous solvent in which powders of MC or KB were dispersed, and the mixed solution was finally reduced with sodium borohydride to grow Pt NPs on the surface of MC or KB. After the supernatant solution was removed by centrifugation, the precipitation products composed of Pt particles on MC(Pt-NP(*w*)/MC) or KB(Pt-NP(*w*)/KB) were further used to fabricate the electrode.

### Electrochemical measurements

The working electrodes were prepared using four types of Pt-NC(*d*)/MC, Pt-NC(*d*)/KB, Pt-NP(*w*)/MC, and Pt-NP(*w*)/KB.^32,33,45^ First, catalyst inks were prepared by mixing 18.5 mg of the carbon-supported catalysts with 19 mL of deionized water, 6.0 mL of 2-propanol, and diluted ionomer (5 wt% Nafion) solution. The amount of Nafion solution was 33 and 100 μL for Pt-NC(NP)/MC and Pt-NC(NP)/KB, respectively. 10 μL of the catalyst ink was dropped onto a Pt disk electrode after cleaning and drying the electrode. The prepared working electrode was used for a triple electrode cell system consisting of a Pt electrode as the counter electrode and Ag/AgCl as the reference electrode. To remove the influence of oxygen, all cyclic voltammetry (CV) measurements were performed in a deoxygenated 0.1 mol L^−1^ HClO_4_ solution. The cathode was purged with nitrogen for 30 min before the CV measurements. The CV curve was measured at room temperature at a scanning rate of 50 mV s^−1^ in the range of −150–1000 mV *versus* Ag/AgCl.

### MEA preparation and cell performance measurement

**Fig. 2:**
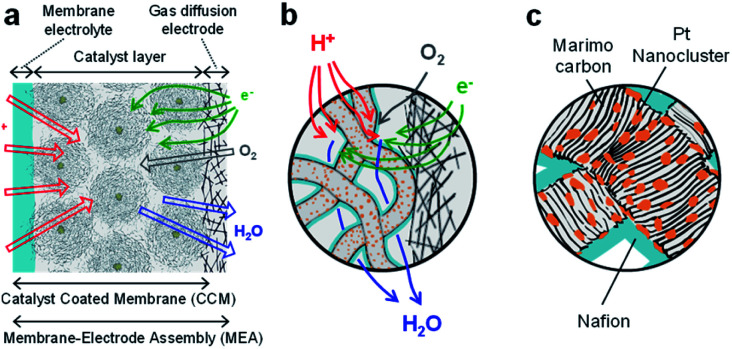
(a) Schematic cross section of Membrane-Electrode Assembly (MEA) which consists of a membrane electrolyte, catalyst layer, and gas diffusion electrode. (b) Expanded schematic view of interface between catalyst layer and gas diffusion electrode in (a). In the catalyst layer, Pt catalysts on Marimo carbon (MC) are used together with Nafion coating for proton transport and leaving H_2_O. (c) Expanded schematic view of Pt NC/NP catalysts on MC in the catalyst layer in (a) and (b).

The MEA electrode was prepared using four types of Pt-NC(*d*)/MC, Pt-NC(*d*)/KB, Pt-NP(*w*)/MC, and Pt-NP(*w*)/KB. To fabricate the catalyst inks, the carbon-supported catalyst of Pt-NC(NP)/MC or Pt-NC(NP)/KB was suspended in water and different amounts of diluted ionomer (5 wt% Nafion) solution were added, forming different ionomer/carbon weight ratios of Nafion to Pt NC(NP) on MC or KB. The weight ratios of water to carbon were 8.5 and 10, and the weight ratios of ionomer to carbon were 0.1 and 0.7 for Pt-NC(NP)/MC and Pt-NC(NP)/KB, respectively. By spraying the various catalyst inks on the membrane electrolyte (80 mm × 80 mm) (Du Pont, Nafion NRE212), catalyst-coated membranes (CCMs) were prepared with an area of 50 mm × 50 mm and were then processed by hot pressing of 47 kg cm^−2^, 155 °C, and 10 min. The membrane electrolyte plays a role of layer material for proton transfer between the anode and cathode, as shown in Fig. 2(a). The amount of catalysts was determined from the amount of Pt NCs against the electrode area of the MEA (0.05 mg_Pt_ cm^−2^) for both anode and cathode. Finally, the MEAs were fabricated by the CCMs in contact with commercially available gas diffusion electrodes (Toray Carbon Paper, TGP-H-60), as illustrated in Fig. 2.

In the evaluation of cell performance using the fabricated MEAs, current and voltage (*I*–*V*) measurements were performed for PEFCs. The *I*–*V* measurements were performed at a cell temperature of 80 °C. Humidified hydrogen gas (200 mL min^−1^, 100% relative humidity (RH) at 80 °C) and humidified air (200 mL min^−1^, 100% RH at 70 °C) were supplied to the anode and cathode, respectively,^45^ and gas utilization is approximately 70% at the anode and 40% at the cathode.

## Results and discussion

### TEM observation of Pt NCs

**Fig. 3:**
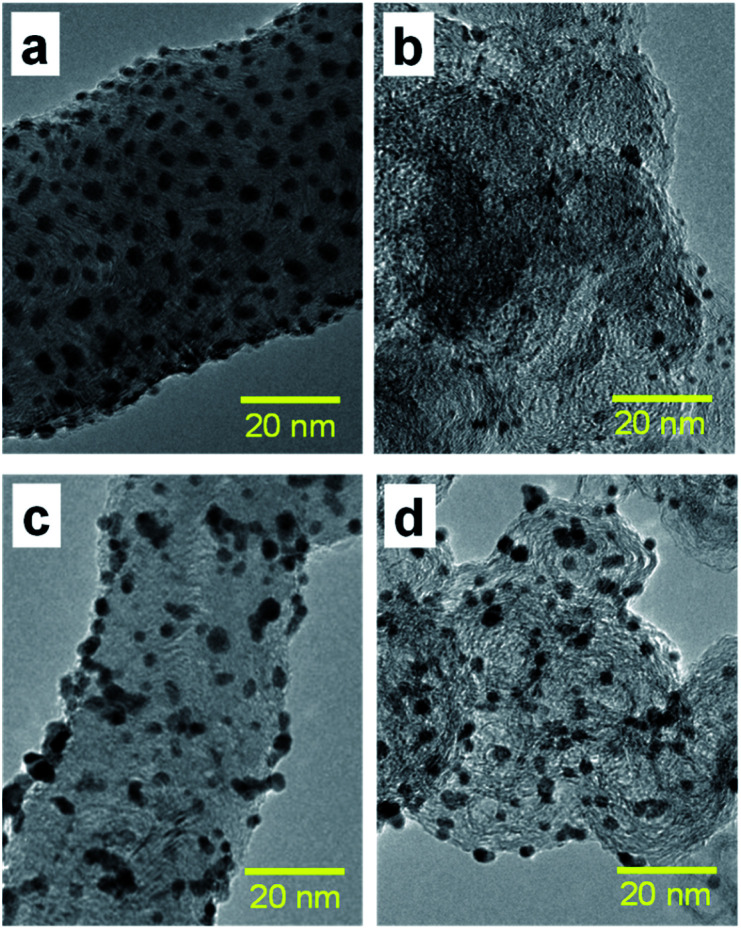
(a) Transmission electron microscopy (TEM) images of Pt catalysts prepared by (a) dry process on Marimo carbon (Pt-NC(*d*)/MC), (b) dry process on carbon black (Ketjenblack; KB, Pt-NC(*d*)/KB), (c) wet process on MC (Pt-NP(*w*)/MC), and (d) wet process on KB (Pt-NP(*w*)/KB). The corresponding size distributions of Pt catalysts are presented in Fig. 4. See also X-ray diffraction patterns (Fig. S1†) and TEM images with large field of view (Fig. S2†) in the ESI.†

**Fig. 4:**
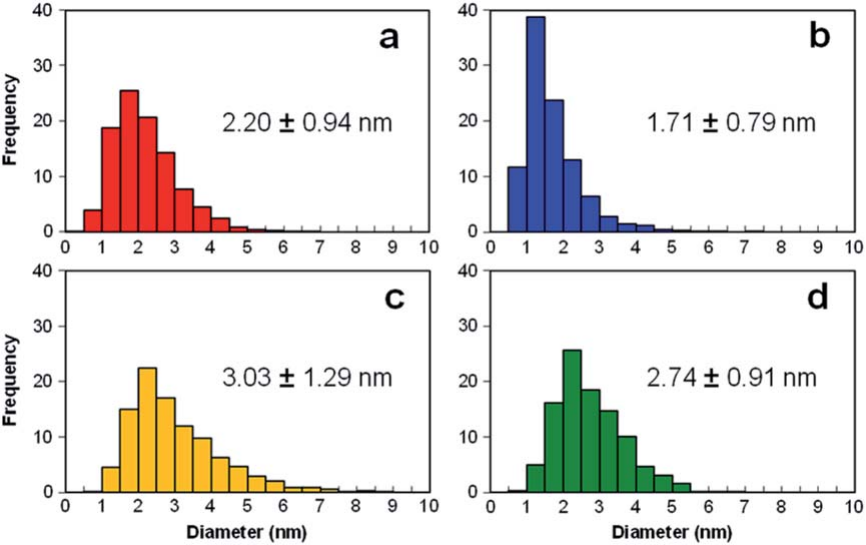
Histograms of diameter distributions of Pt catalysts prepared by (a) dry process on Marimo carbon (Pt-NC(*d*)/MC), (b) dry process on carbon black (Ketjenblack; KB, KB (Pt-NC(*d*)/KB)), (c) wet process on MC (Pt-NP(*w*)/MC), and (d) wet process on KB (Pt-NP(*w*)/KB), which correspond to the TEM images in Fig. 3.

Fig. 3(a)–(d) present the TEM images of Pt catalysts prepared by (a) the dry process on MC, (b) the dry process on KB, (c) the wet process on MC, and (d) the wet process on KB, respectively, which are expressed as Pt-NC(*d*)/MC, Pt-NC(*d*)/KB, Pt-NP(*w*)/MC, and Pt-NP(*w*)/KB, respectively. The contents of Pt NCs/NPs were evaluated to be (a) 8.4 wt%, (b) 8.5 wt%, (c) 6.0 wt% and (d) 8.5 wt%, respectively, *via* thermogravimetry (TG) analysis. The diameter distributions of the Pt NCs were evaluated based on 100 particles in randomly selected TEM images and are illustrated in the histograms in Fig. 4(a)–(d). We first compare the diameter distribution features in Pt loading in the gas phase and liquid phase and then, we compare their features in MC and KB supports.

By comparing the diameter distributions of Pt catalysts in the gas phase and liquid phase, the histograms in Fig. 4 indicate that the average diameter of Pt NCs synthesized in the gas phase is approximately 1 nm smaller than that of Pt NPs synthesized in the liquid phase: similar size differences could be observed in Fig. 4(a) and (c) on MC and Fig. 4(b) and (d) on KB. Because the reported sizes of Pt catalysts synthesized in the liquid phase^32,45^ are similar to or larger than the size in this study, the results indicate that the DiPET method enables us to fabricate a smaller Pt catalyst on carbon material supports.

Furthermore, the widths of the distribution (standard deviation) of Pt NCs synthesized in gas-phase DiPET are smaller than those of Pt NPs synthesized in the liquid phase. Although NC/NP growth can be characterized by Pt atom aggregation either in the gas phase or liquid phase, aggregation in the gas phase may occur more uniformly than that in the liquid phase because the aggregation in the liquid is a rather inhomogeneous process through chemical reduction reactions.^42,43^ Treatments involving no chemical reagents or solvents appear to yield a cleaner Pt catalyst in the dry process; dry-deposition Pt catalysts.

A comparison of the size distributions on MC and KB demonstrates that both generation methods yield about 0.3 to 0.5 nm larger Pt NC/NP on MC than those on KB. Comparing Fig. 4(a) and (b) with DiPET and Fig. 4(c) and (d) with the wet process, the distributions are similar. Although it is not easy to extract the formation mechanism for Pt NCs/NPs, the size difference may be related to the nature of the site for Pt catalyst immobilization. For MC, the immobilization site is an edge of stacked graphene sheets, where carbon nanofilaments of MC grow radially from diamond oxide in a stacked cup shape.^32,45^ The stacked graphene edges appear to exhibit high selectivity for immobilizing Pt catalysts. For KB, in contrast, the immobilization sites of the mainly hexagonal structure of the graphite surface on KB^23^ is not as active as the edge of the stacked graphene in MC, because KB has a porous carbon structure. In fact, the shapes of Pt-NC(*d*)/KB and Pt-NP(*w*)/KB appear almost spherical (non-deformed) in the TEM images.

It should be noted that the TEM images of Pt-NC(*d*)/MC and Pt-NP(*w*)/MC display an ellipsoidal shape, where Pt NCs/NPs are slightly crushed in the direction perpendicular to the support surface, implying close interaction with the edge of the stacked graphene in MC.

### Electrochemical measurements

**Fig. 5:**
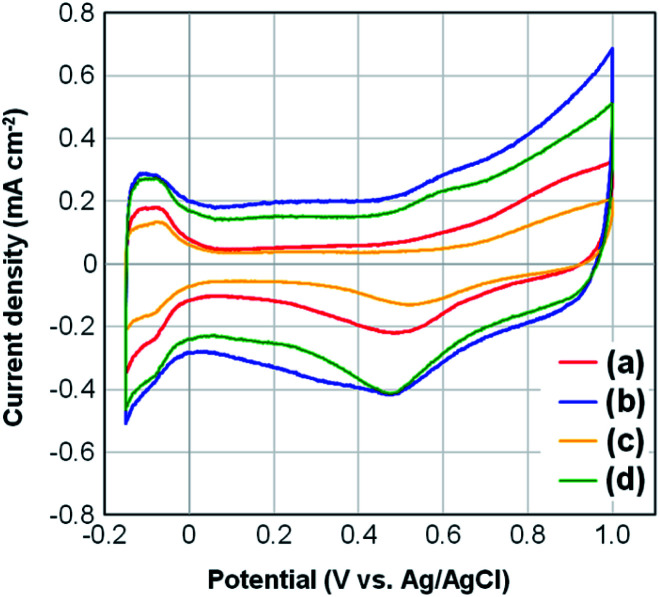
Changes in cyclic voltammograms for (a) Pt (dry process) on Marimo carbon (MC), (b) Pt (dry process) on Ketjenblack (KB), (c) Pt (wet process) on MC, and (d) Pt (wet process) on KB, which correspond to those in Fig. 3 and 4.

Fig. 5 presents the CV curves for four samples of Pt-NC(*d*)/MC, Pt-NC(*d*)/KB, Pt-NP(*w*)/MC, and Pt-NP(*w*)/KB. The contents of Pt NCs estimated by TG analysis were (a) 8.4, (b) 8.5, (c) 6.0, and (d) 8.5 wt%, respectively. All curves include peaks originating from hydrogen adsorption and desorption in the region of −0.15–0.0 V. The lower peak observed in the region of 0.2–0.6 V originates from the reduction of Pt NCs. The electrochemically active surface area (ECSA) (m^2^ g^−1^) of Pt was calculated from the hydrogen adsorption peak area of the CV curves (*Q*_Had_) (C) as follows:^46^

1 ECSA = *Q*_Had_/(2.1 × [Pt])

where 2.1 (C m^−2^) is the constant factor conventionally used for clean polycrystalline Pt, and [Pt] (g) is the weight of Pt loaded on the electrode. From Fig. 5, the ECSA values were calculated as (a) 35.0 m^2^ g^−1^, (b) 26.2 m^2^ g^−1^, (c) 37.3 m^2^ g^−1^, and (d) 31.4 m^2^ g^−1^. A comparison between Pt-NC(NP) on MC (Fig. 5(a) and (c)) and Pt-NC(NP) on KB (Fig. 5(b) and (d)) indicates that the ECSA values for Pt-NC(NP) on MC are larger than those on KB. The results are discussed below from a morphological perspective using the TEM images.

The larger ECSA values for Pt-NC(NP)/MC can be ascribed to the immobilization site. The Pt NCs/NPs are principally supported on the edge of stacked graphene sheets, and much fewer Pt NCs/NPs are supported in internal pores. The edge site can efficiently contribute to increase the electrochemical activity owing to a sparse space for reactant gases. Moreover, flattening deformation due to the interaction at the graphene edge may also contribute to increasing the active surface area.

In contrast, the KB support allows the Pt NCs/NPs to be supported deep within the internal pores, and the buried Pt NCs/NPs may be electrochemically inactivated, causing a decrease in ECSA.^44^ Although porous carbon materials, such as KB, are often used for support for Pt catalysts to increase the supported area, only some Pt catalysts supported on the outer surface seem to exhibit electrochemical activity. In particular, Pt-NC(*d*)/KB synthesized in the gas phase exhibits the lowest ECSA value among the four samples, which can be attributed to a high ratio of buried Pt NCs immobilized in the internal pores of KB,^35^ as smaller NCs synthesized in the gas phase can enter the pores more efficiently. Note that the amount of ionomers is also related to the porosity of the electrocatalysts used in PEFCs, and the specific surface areas before and after the addition of Nafion to the catalysts are shown in Fig. S3† in the ESI.†

### Single cell performance of Pt NCs

**Fig. 6:**
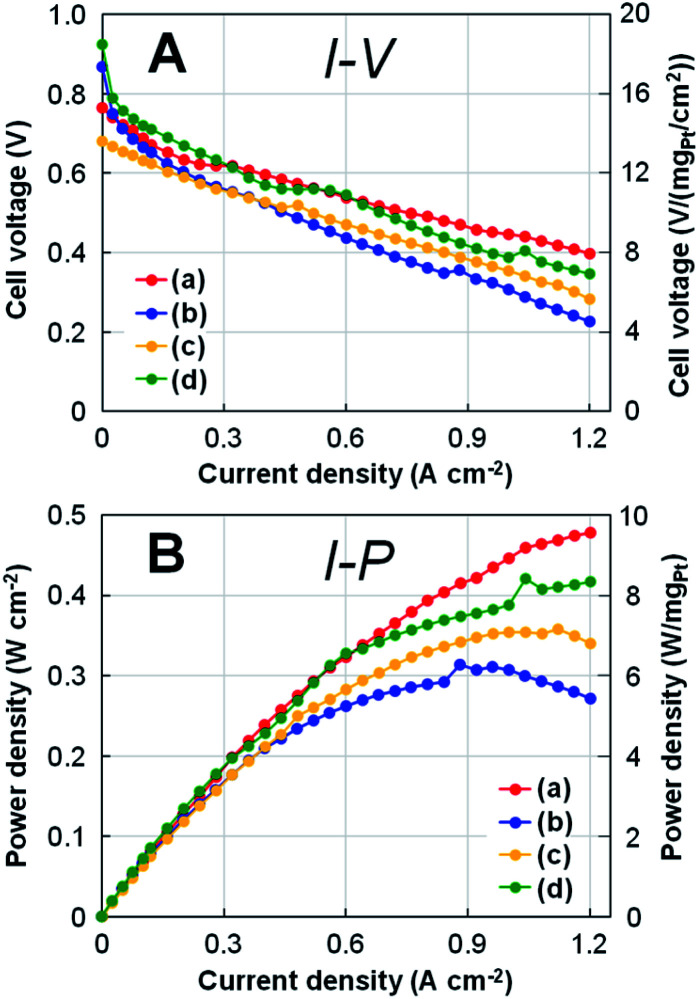
(A) Polarization *I*–*V* curves and (B) power density *I*–*P* curves for a single cell with the MEAs at Pt loading of 0.05 mg_Pt_ cm^−2^ for both anode and cathode with (a) Pt (dry process) on MC (Pt-NC(*d*)/MC), (b) Pt (dry process) on KB (Pt-NC(*d*)/KB), (c) Pt (wet process) on MC (Pt-NP(*w*)/MC), and (d) Pt (wet process) on KB (Pt-NP(*w*)/KB), which correspond to those in Fig. 3, 4, and 5. On the right side of each panel, the values of cell voltage and power density per Pt loading (0.05 mg_Pt_ cm^−2^) are shown.

Fig. 6 shows the results of (A) polarization *I*–*V* and (B) power density *I*–*P* curves for four types of Pt-NC(*d*)/MC, Pt-NC(*d*)/KB, Pt-NP(*w*)/MC, and Pt-NP(*w*)/KB at Pt loading of 0.05 mg_Pt_ cm^−2^ for both anode and cathode MEAs. Comparing Pt-NC(*d*)/MC and Pt-NC(*d*)/KB at a current density of 1.2 A cm^−2^, the decrease in cell voltage of Pt-NC(*d*)/MC against the current density is suppressed to less than 50% of the initial cell voltage (0.77 → 0.40 V), whereas Pt-NC(*d*)/KB exhibits a large decrease in the cell voltage (more than 60%; 0.87 → 0.23 V). Relatively low performance in Pt-NC(*d*)/MC is attributed to overpotential due to ohmic resistance between rolled graphene sheets, where electron transfer rates are lowered. Although Pt-NC(*d*)/MC exhibits a lower cell voltage than that of Pt-NP(*w*)/KB up to a current density of 0.5 A cm^−2^, the cell voltage of Pt-NC(*d*)/MC becomes larger than the others above 0.6 A cm^−2^; in the *I*–*P* curve, Pt-NC(*d*)/MC shows the highest power output density in the high current density region.

More quantitatively, the single cell performance is obtained by the minimum Pt loading of 0.05 mg_Pt_ cm^−2^ for both anode and cathode MEAs: at a current density of 1.2 A cm^−2^, Pt-NC(*d*)/MC MEAs show a cell voltage of 0.40 V and power density of 9.6 W cm^−2^, as shown in Fig. 6. When converted to Pt loading amount, these values are 8.0 V (mg_Pt_ cm^−2^)^−1^ and 0.105 g_Pt_ kW^−1^ (power density (rated)). Although similar small power density (rated) of around 0.1 g_Pt_ kW^−1^ have been recently reported for other studies of activated carbon composite Pt catalyst^47^ and silica-decorated carbon-Pt electrocatalyst^48^ at the current density of 1.2 A cm^−2^, the cell voltage per Pt loading for Pt-NC(*d*)/MC of 8.0 V (mg_Pt_ cm^−2^)^−1^ is better than any other measurement.

Finally, comparing Pt-NC(*d*)/MC synthesized in the gas phase and Pt-NP(*w*)/MC synthesized in the liquid phase reveals that Pt-NC(*d*)/MC exhibits better power generation than Pt-NP(*w*)/MC. This is likely because the Pt catalysts of Pt-NC(*d*)/MC are smaller in size and more evenly dispersed spatially than those of Pt-NP(*w*)/MC, thereby improving the power generation performance. Since the working reaction gases of H_2_ and O_2_ must efficiently enter the catalyst layer in the FC, a sparse space for gas flow is generally important for designing nanostructures.^49^ In particular, leaving the generated water outside the catalytic layer becomes more crucial in the high current density region. The better performance of Pt-NC(*d*)/MC at a high current density of 1.2 A cm^−2^ shows that Pt NC(*d*)/MC is more effective for the discharge of generated water for a single cell with 0.05 mg_Pt_ cm^−2^ MEAs.

Importantly, the improved output with Pt-NC(*d*)/MC survives even at 1000 cycles as higher than 0.2 V at 0.6 A cm^−2^, whereas the output with Pt-NC(*d*)/KB rapidly deteriorates; a cell voltage of 0.2 V is output only at 0.45 A cm^−2^ or less after 500 cycles (see Fig. S4 in the ESI†). Therefore, it can be concluded that the combination of Pt NCs and MC by gas phase synthesis enables the development of a more efficient FC by well-designed nanostructures effectively even with a less amount of Pt catalyst loading.

## Conclusions

In this study, to increase the Pt utilization efficiency using a DiPET method, smaller and uniform size of naked Pt NC catalysts generated in the gas phase were supported on MC powder that comprise a high density of carbon nanofilaments, forming Pt-NC(*d*)/MC. An MEA with a size of 25 cm^2^ size was formed by the dry-deposition of Pt-NC(*d*)/MC, and at minimum Pt loading of 0.05 mg_Pt_ cm^−2^ for both anode and cathode, and a single cell exhibited superior power density (rated) of 0.105 g_Pt_ kW^−1^ at a current density of 1.2 A cm^−2^, which is better output power density than the conventional MEA formed by Pt NC catalysts on MC *via* a liquid-phase synthesis. Based on the output efficiency obtained using Pt NC/NP catalysts on other carbon black nanomaterials of KB, the FC performance with Pt-NC(*d*)/MC was particularly improved in the high current density region above 0.6 A cm^−2^. By TEM measurements, the improved performance was attributed to the fact that Pt catalysts that are spatioselectively and monodispersely loaded on the outer surface of MC can facilitate not only the gas reaction but also leaving the generated water. The dry fabrication processes in both Pt NC formation and MC formation, including powder trapping, provide a superior approach for improving the Pt utilization efficiency by optimizing surface immobilization and nanostructures morphologically. It is possible to scale up the production of the dry-deposition Pt-NC(*d*)/MC by expanding the irradiation area of the NC beam; (1) target shape is changed from a flat disk target (Pt or Pt alloy) to a cylindrical target and (2) beam shape is changed from a small circle to a long slot. This study illustrated key features of NC vapor deposition on MC using the dry method of DiPET with a pure Pt catalyst, and moreover, this method is applicable to (i) naked Pt alloy catalysts with an alloy sputtering target or with multiple sputtering sources and (ii) various powder materials for a catalyst support, leading to better FC performance.

## Author contributions

K. T., M. E., T. A., and A. N. supervised the project. N. H., M. T., K. T., and A. N. carried out the dry deposition catalyst experiments. Y. K., H. G., and M. E. carried out the wet process catalyst and fuel cell experiments. T. A. performed transmission electron microscopy measurements. All authors discussed the results and assisted during manuscript preparation.

## Conflicts of interest

The authors declare the following competing financial interest(s): K. T. and A. N. are inventors on JAPAN patent JP 5493139, submitted by JST agency and Ayabo Corp., which covers nanocluster generator.

## Supplementary Material

RA-011-D1RA07717A-s001

## References

[cit1] Chow J., Kopp R. J., Portney P. R. (2003). Science.

[cit2] Gasteiger H. A., Markovic N. M. (2009). Science.

[cit3] Stamenkovic V. R., Mun B. S., Arenz M., Mayrhofer K. J. J., Lucas C. A., Wang G., Ross P. N., Markovic N. M. (2007). Nat. Mater..

[cit4] Koh S., Strasser P. (2007). J. Am. Chem. Soc..

[cit5] Shao M. H., Shoemaker K., Peles A., Kaneko K., Protsailo L. (2010). J. Am. Chem. Soc..

[cit6] Wang D. L., Xin H. L. L., Hovden R., Wang H. S., Yu Y. C., Muller D. A., DiSalvo F. J., Abruna H. D. (2013). Nat. Mater..

[cit7] Guo S., Li D., Zhu H., Zhang S., Markovic N. M., Stamenkovic V. R., Sun S. (2013). Angew. Chem., Int. Ed..

[cit8] Chen C., Kang Y., Huo Z., Zhu Z., Huang W., Xin H. L., Snyder J. D., Li D., Herron J. A., Mavrikakis M., Chi M., More K. L., Li Y., Markovic N. M., Somorjai G. A., Yang P., Stamenkovic V. R. (2014). Science.

[cit9] Han B., Carlton C. E., Kongkanand A., Kukreja R. S., Theobald B. R., Gan L., O'Malley R., Strasser P., Wagner F. T., Shao-Horn Y. (2015). Energy Environ. Sci..

[cit10] Zhao X., Chen S., Fang Z. C., Ding J., Sang W., Wang Y. C., Zhao J., Peng Z. M., Zeng J. (2015). J. Am. Chem. Soc..

[cit11] Escudero-Escribano M., Malacrida P., Hansen M. H., Vej-Hansen U. G., Velazquez-Palenzuela A., Tripkovic V., Schiotz J., Rossmeisl J., Stephens I. E., Chorkendorff I. (2016). Science.

[cit12] Wang R., Wang H., Luo F., Liao S. (2018). Electrochem. Energy Rev..

[cit13] Banham D., Choi J. Y., Kishimoto T., Ye S. Y. (2019). Adv. Mater..

[cit14] Kong F., Ren Z., Banis M. N., Du L., Zhou X., Chen G., Zhang L., Li J., Wang S., Li M., Doyle-Davis K., Ma Y., Li R., Young A., Yang L., Markiewicz M., Tong Y., Yin G., Du C., Luo J., Sun X. (2020). ACS Catal..

[cit15] Ren X., Lv Q., Liu L., Liu B., Wang Y., Liu A., Wu G. (2020). Sustainable Energy Fuels.

[cit16] Sapkota P., Boyer C., Dutta R., Cazorla C., Aguey-Zinsou K.-F. (2020). Sustainable Energy Fuels.

[cit17] Debe M. K. (2012). Nature.

[cit18] Chen S., Sheng W., Yabuuchi N., Ferreira P. J., Allard L. A., Shao-Horn Y. (2009). J. Phys. Chem. C.

[cit19] Oezaslan M., Hasché F., Strasser P. (2012). J. Electrochem. Soc..

[cit20] Stephens I. E. L., Bondarenko A. S., Grønbjerg U., Rossmeisl J., Chorkendorff I. (2012). Energy Environ. Sci..

[cit21] Garlyyev B., Pohl M. D., Čolić V., Liang Y., Butt F. K., Holleitner A., Bandarenka A. S. (2018). Electrochem. Commun..

[cit22] Zalitis C. M., Kramerw D., Kucernak A. R. (2013). Phys. Chem. Chem. Phys..

[cit23] Holade Y., Morais C., Servat K., Napporn T. W., Kokoh K. B. (2014). Phys. Chem. Chem. Phys..

[cit24] Roy C., Knudsen B. P., Pedersen C. M., Velázquez-Palenzuela A., Christensen L. H., Damsgaard C. D., Stephens I. E. L., Chorkendorff I. (2018). ACS Catal..

[cit25] Barbosa E. C. M., Parreira L. S., de Freitas I. C., Aveiro L. R., de Oliveira D. C., dos Santos M. C., Camargo P. H. C. (2019). ACS Appl. Energy Mater..

[cit26] Fichtner J., Watzele S., Garlyyev B., Kluge R. M., Haimerl F., El-Sayed H. A., Li W.-J., Maillard F. M., Dubau L., Chattot R., Michalička J., Macak J. M., Wang W., Wang D., Gigl T., Hugenschmidt C., Bandarenka A. S. (2020). ACS Catal..

[cit27] Okonkwo P. C., Otor C. (2021). Int. J. Energy Res..

[cit28] Trogadas P., Cho J. I. S., Kapil N., Rasha L., Corredera A., Brett D. J. L., Coppens M.-O. (2020). Sustainable Energy Fuels.

[cit29] Zhang Z., Jiang C., Li P., Feng Q., Zhao Z. l., Yao K., Fan J., Li H., Wang H. (2021). Sustainable Energy Fuels.

[cit30] Huang Q., Guo Y., Chen D., Zhang L., Li T.-T., Hu Y., Qian J., Huang S. (2021). Chem. Eng. J..

[cit31] Chen D., Han C., Sun Q., Ding J., Huang Q., Li T.-T., Hu Y., Qian J., Huang S. (2021). Green Energy Environ..

[cit32] Eguchi M., Okubo A., Yamamoto S., Kikuchi M., Uno K., Kobayashi Y., Nishitani-Gamo M., Ando T. (2010). J. Power Sources.

[cit33] Eguchi M., Baba K., Onuma T., Yoshida K., Iwasawa K., Kobayashi Y., Uno K., Komatsu K., Kobori M., Nishitani-Gamo M., Ando T. (2012). Polymers.

[cit34] Nakagawa K., Oda H., Yamashita A., Okamoto M., Sato Y., Gamo H., Nishitani-Gamo M., Ogawa K., Ando T. (2009). J. Mater. Sci..

[cit35] Sui S., Wang X., Zhou X., Su Y., Riffat S., Liu C.-j. (2017). J. Mater. Chem. A.

[cit36] Jayawickrama S. M., Fujigaya T. (2021). J. Power Sources.

[cit37] Nakaya M., Iwasa T., Tsunoyama H., Eguchi T., Nakajima A. (2014). Adv. Funct. Mater..

[cit38] Tsunoyama H., Zhang C., Akatsuka H., Sekiya H., Nagase T., Nakajima A. (2013). Chem. Lett..

[cit39] Zhang C., Tsunoyama H., Akatsuka H., Sekiya H., Nagase T., Nakajima A. (2013). J. Phys. Chem. A.

[cit40] Stephens I. E. L., Rossmeisl J., Chorkendorff I. (2016). Science.

[cit41] Yarlagadda V., Carpenter M. K., Moylan T. E., Kukreja R. S., Koestner R., Gu W., Thompson L., Kongkanand A. (2018). ACS Energy Lett..

[cit42] Tsunoyama H., Ohnuma A., Takahashi K., Velloth A., Ehara M., Ichikuni N., Tabuchi M., Nakajima A. (2019). Chem. Commun..

[cit43] Tsunoyama H., Akatsuka H., Shibuta M., Iwasa T., Mizuhata Y., Tokitoh N., Nakajima A. (2017). J. Phys. Chem. C.

[cit44] Tsunoyama H., Shibuta M., Nakaya M., Eguchi T., Nakajima A. (2018). Acc. Chem. Res..

[cit45] Baba K., Nishitani-Gamo M., Ando T., Eguchi M. (2016). Electrochim. Acta.

[cit46] Larminie J. and Dicks A., Fuel Cell Systems Explained, Wiley, Chichester, UK, 2nd edn, 2003

[cit47] Kim T., Xie T., Jung W., Gadala-Maria F., Ganesan P., Popov B. N. (2015). J. Power Sources.

[cit48] Dhanasekaran P., Shukla A., Selvaganesh S. V., Mohan S., Bhat S. D. (2019). J. Power Sources.

[cit49] Padgett E., Andrejevic N., Liu Z., Kongkanand A., Gu W., Moriyama K., Jiang Y., Kumaraguru S., Moylan T. E., Kukreja R., Muller D. A. (2018). J. Electrochem. Soc..

