# Supplementary material for: Platinum nanocluster catalysts supported on Marimo carbon via scalable dry deposition synthesis
Source: RSC Adv. 2021 Dec 8;11(62):39216–22. doi: 10.1039/d1ra07717a (PMC9044432; doi:10.1039/d1ra07717a)
Supplement: RA-011-D1RA07717A-s001 [file RA-011-D1RA07717A-s001.pdf]

Electronic Supplementary Material (ESI) for RSC Advances.
This journal is © The Royal Society of Chemistry 2021

Supplementary Information for

Scalable Dry Deposition Synthesis of Platinum Nanocluster Catalysts Immobilized on Marimo Carbon for Polymer Electrolyte Fuel Cell

Naoyuki Hirata,[†] Yui Katsura,[‡] Hiroyuki Gunji,[‡] Masahide Tona,[†] Keizo Tsukamoto,[†,*] Mika Eguchi,[‡,*] Toshihiro Ando,[⊥,*] and Atsushi Nakajima[§,*]

[†]*Ayabo Co., Ltd., 1 Hosogute, Fukukama-cho, Anjo, Aichi 446-0052, Japan*

[‡] *College of Engineering, Ibaraki University, 4-12-1 Nakanarusawa, Hitachi, Ibaraki 316-8511, Japan*

[§] *Department of Chemistry, Keio University, 3-14-1 Hiyoshi, Kohoku-ku, Yokohama, Kanagawa 223-8522, Japan*

[⊥] *National Institute for Materials Science, Tsukuba, Ibaraki 305-0044, Japan*

*Address correspondence to K. Tsukamoto, M. Eguchi, T. Ando, A. Nakajima
E-mails: (K. Tsukamoto) keizo@ayabo.com; (M. Eguchi) mika.eguchi.m@vc.ibaraki.ac.jp; (T. Ando) ANDO.Toshihiro@nims.go.jp; A. Nakajima (A. Nakajima) nakajima@chem.keio.ac.jp

Table of contents

Figure S1. XRD patterns for Pt-NC(*d*)/MC, Pt-NC(*d*)/KB, Pt-NP(*w*)/MC, and Pt-NP(*w*)/KB. **(S2)**
Figure S2. TEM images for Pt-NC(*d*)/MC, Pt-NC(*d*)/KB, Pt-NP(*w*)/MC, and Pt-NP(*w*)/KB. **(S2)**
Figure S3. The specific surface areas of the catalysts by BET surface area analysis. **(S3)**
Figure S4. Cycle dependent *I-V* curves for the single cell for Pt-NC(*d*)/MC and Pt-NC(*d*)/KB. **(S3)**

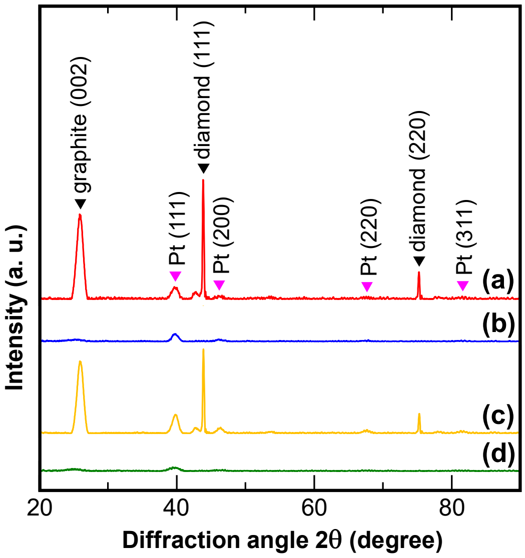

Figure S1. X-ray diffraction (XRD) patterns for (a) dry process on Marimo carbon (Pt-NC(*d*)/MC), (b) dry process on carbon black (Ketjenblack; KB, KB (Pt-NC(*d*)/KB)), (c) wet process on MC (Pt-NP(*w*)/MC), and (d) wet process on KB (Pt-NP(*w*)/KB), which correspond to the TEM images in Figs. 3 and S2, and the diameter histograms in Fig. 4. They were measured with XRD system of Ultima IV, Rigaku Corporation. Diffraction peaks centered at 43.9° and 75.3° could be indexed to (111) and (220) planes of diamond derived from Ni/O–diamond of a catalyst for MC synthesis. Diffraction peak centered at 26.0° could be indexed to (002) plane of graphite derived from carbon nanofilaments in MC. The diamond and graphite peaks were not observed for the catalysts using KB. In all XRD patterns, four broad peaks were observed at 39.9°, 46.4°, 67.7°, and 81.5°, assignable to (111), (200), (220), and (311) of the face-centered-cubic (fcc) Pt.

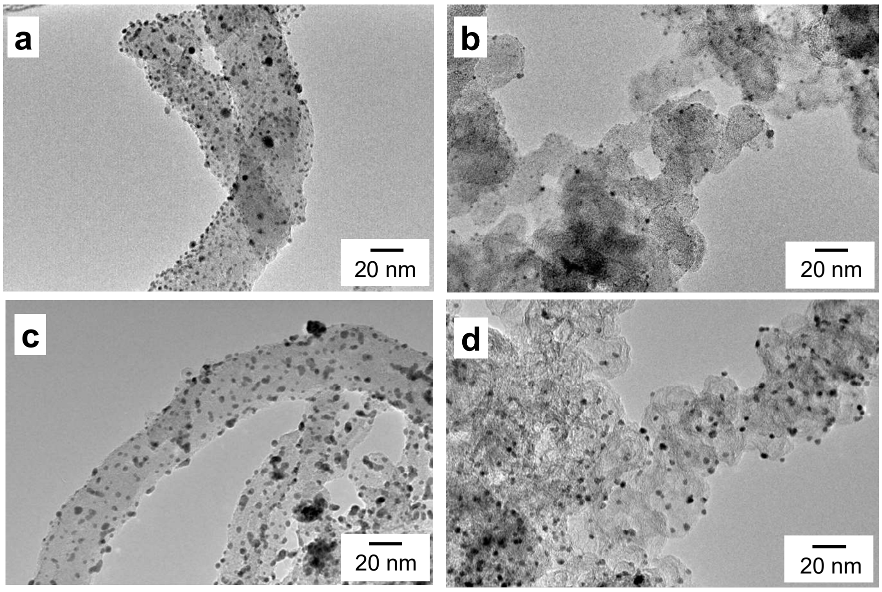

Figure S2. Transmission electron microscopy (TEM) images with large field of view for (a) dry process on Marimo carbon (Pt-NC(*d*)/MC), (b) dry process on carbon black (Ketjenblack; KB, KB (Pt-NC(*d*)/KB)), (c) wet process on MC (Pt-NP(*w*)/MC), and (d) wet process on KB (Pt-NP(*w*)/KB), which correspond to other TEM images in Fig. 3, XRD patterns in Fig. S1, and the diameter histograms in Fig. 4.

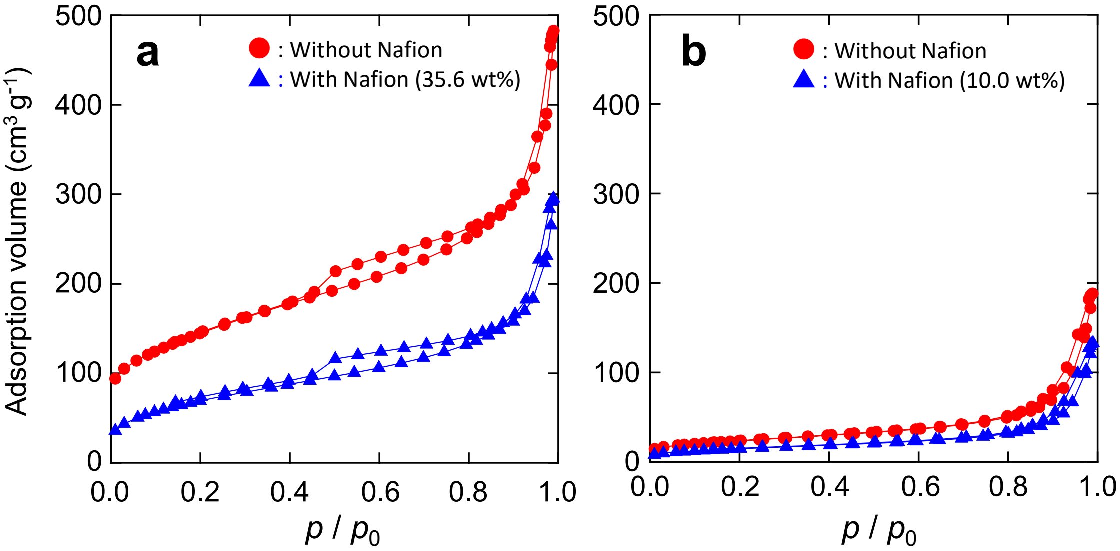

Figure S3. The specific surface areas that are relevant to porosity and pore information were evaluated the nitrogen adsorption isotherms by the Brunauer-Emmett-Teller (BET) surface area analysis; before and after the addition of Nafion to catalysts. The specific surface area after adding Nafion correspond to the specific surface area when the electrode is fabricated for polymer electrolyte FCs. For KB, specific surface areas are 502 and 249 m^2g^{-1} before and after the addition of Nafion to Pt-NP(*w*)/KB catalyst, where the amount of Nafion required for catalyst ink is 35.6 wt%. On the other hand, for MC, they are 82 and 52 m^2g^{-1} before and after the addition of Nafion to Pt-NP(*w*)/MC catalyst, where the amount of Nafion required for catalyst ink is 10.0 wt%. While a porous support generally makes it easier for gas diffusion, it also requires the more ionomer, resulting in decrease in the space volume.

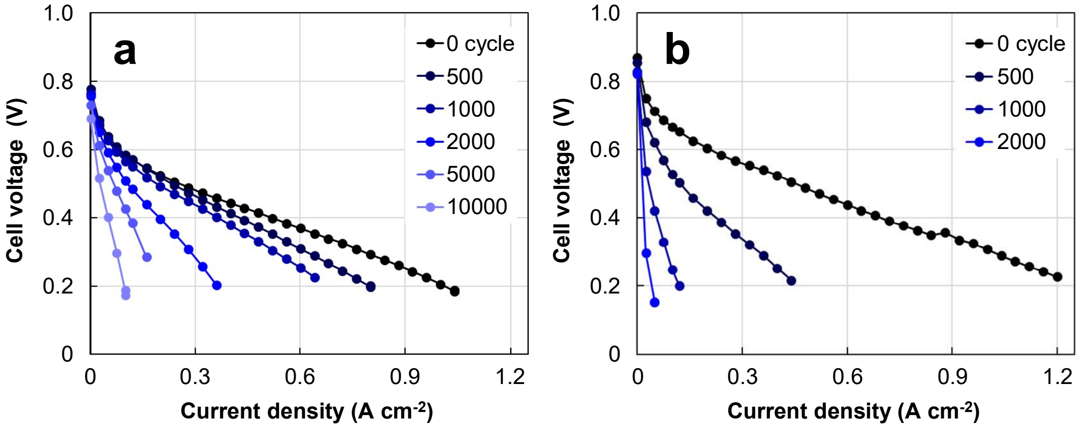

Figure S4. Cycle dependent changes in *I-V* curves for the single cell with MEAs of (a) Pt-NCs on MC (Pt-NC(*d*)/MC) at 0-10000 cycles and (b) Pt-NCs on KB (Pt-NC(*d*)/KB) at 0-2000 cycles. With Pt-NC(*d*)/MC in (a), a superior output greatly survives.
